# Enhancing Geomagnetic Navigation with PPO-LSTM: Robust Navigation Utilizing Observed Geomagnetic Field Data

**DOI:** 10.3390/s25123699

**Published:** 2025-06-13

**Authors:** Xiaohui Zhang, Wenqi Bai, Jun Liu, Songnan Yang, Ting Shang, Haolin Liu

**Affiliations:** School of Automation and Information Engineering, Xi’an University of Technology, Xi’an 710048, China; xhzhang@xaut.edu.cn (X.Z.); liujun0310@xaut.edu.cn (J.L.); yang.son.nan@stu.xaut.edu.cn (S.Y.); st107@xaut.edu.cn (T.S.); haolinliu@xaut.edu.cn (H.L.)

**Keywords:** deep reinforcement learning, partially observed Markov decision process, bionic geomagnetic navigation, proximal policy optimization

## Abstract

Geospatial navigation in GPS-denied environments presents significant challenges, particularly for autonomous vehicles operating in complex, unmapped regions. We explore the Earth’s geomagnetic field, a globally distributed and naturally occurring resource, as a reliable alternative for navigation. Since vehicles can only observe the geomagnetic field along their traversed paths, they must rely on incomplete information to infer the navigation strategy; therefore, we formulate the navigation problem as a partially observed Markov decision process (POMDP). To address this POMDP, we employ proximal policy optimization with long short-term memory (PPO-LSTM), a deep reinforcement learning framework that captures temporal dependencies and mitigates the effects of noise. Using real-world geomagnetic data from the international geomagnetic reference field (IGRF) model, we validate our approach through experiments under noisy conditions. The results demonstrate that PPO-LSTM outperforms baseline algorithms, achieving smoother trajectories and higher heading accuracy. This framework effectively handles the uncertainty and partial observability inherent in geomagnetic navigation, enabling robust policies that adapt to complex gradients and offering a robust solution for geospatial navigation.

## 1. Introduction

### 1.1. Background and Motivation

The geomagnetic field is generated by multiple contributing sources, including both internal and external factors. The core field, commonly referred to as the main field, along with the crustal or lithospheric field, represents the primary internal sources. In contrast, external contributions arise from ionospheric and magnetospheric currents [1]. As a naturally occurring and globally distributed phenomenon, the geomagnetic field exhibits unique characteristics that make it an effective information source for applications, such as navigation [2], positioning [3], and mineral exploration [4]. Its spatially varying yet regionally distinctive properties provide a consistent and reliable reference frame, enabling location-specific orientation without dependence on external infrastructure, with the characteristics of strong autonomy, good concealment, and unrestricted by area and time [5,6,7]. Recent studies have shown that various creatures, such as birds [8], turtles [9], salmon [10], and lobsters [11], navigate by sensing the Earth’s magnetic field. For instance, birds have been found to use the vector direction and inclination of the geomagnetic field for orientation, allowing them to undertake long migratory journeys with remarkable precision [8]. Similarly, research on turtles has revealed that hatchlings rely on a combination of visual, wave, and geomagnetic cues to navigate offshore, while adult turtles develop a learned understanding of geographic magnetic patterns to locate specific targets [9].

These findings have enhanced our understanding of animal orientation and navigation mechanisms [12]. It is unlikely that animals store complete geomagnetic maps in their brains, providing a biological basis for navigation without relying on prior geomagnetic maps [13]. This biological insight has inspired the development of bionic geomagnetic navigation strategies, which mimic natural navigation abilities to enable robust and adaptive navigation in complex environments.

### 1.2. Literature Review

Liu et al. conceptualized bionic geomagnetic navigation as an autonomous search for paths shaped by geomagnetic environmental influences. They proposed a multi-objective navigation framework that integrated a random walk movement model with a stress evolution algorithm [14], providing foundational insights into biologically inspired navigation. However, the generated trajectories lacked stability and consistency, reducing their effectiveness for practical applications. To address these limitations, Liu et al. introduced a genetic algorithm to enhance trajectory [15]. This approach produced smoother paths compared to the stress evolution algorithm [14], but the trajectories remained suboptimal, particularly in environments with complex geomagnetic variations. Building on these efforts, Zhou et al. developed an algorithm based on differential evolution (DE) to enhance search and navigation efficiency [16]. By optimizing the mutation mechanism, the algorithm produced higher-quality solutions. Simulations demonstrated its effectiveness in guiding Autonomous Underwater Vehicles to their destinations, surpassing conventional evolutionary algorithms in terms of trajectory length, computational efficiency, and navigation accuracy. Despite these advancements, heuristic algorithms share a common limitation: the inefficiency of the population fitness evaluation process. Each fitness estimation necessitates that the vehicle execute a navigation action, often leading to the generation of numerous suboptimal trajectories. Furthermore, premature convergence—a persistent challenge in heuristic optimization—introduces additional complications. When navigating areas with significant variations in magnetic field distribution, the vehicle may fail to detect these changes and continue to rely on outdated navigation actions, ultimately resulting in the failure to reach the intended destination. To avoid random searches, Zhou et al. proposed a geomagnetic gradient-based bionic navigation method grounded in the principle of multiparameter simultaneous convergence [17]. Their approach calculates the heading angle using a least-squares approximation method with geomagnetic gradient data, thereby eliminating the need for the random search behaviors typical of heuristic algorithms. As a result, the trajectories exhibit significantly improved smoothness compared to those generated by heuristic methods. However, inaccuracies in the predicted heading angles, arising from the variable nature of geomagnetic field patterns under the multiparameter simultaneous convergence principle, cause the trajectories to deviate notably from the optimal path. These deviations are particularly pronounced in regions with abrupt geomagnetic variations, which limits the method’s overall reliability and accuracy.

Zhang et al. extended this concept by integrating evolutionary algorithms with heading angles predicted based on the multiparameter simultaneous convergence principle, resulting in a geomagnetic gradient-assisted evolutionary algorithm for long-range underwater navigation [13]. In this approach, heading angles derived from geomagnetic gradients were used to constrain the sample space of the evolutionary algorithm, optimizing navigation paths. Additionally, the evaluation function was refined to align with the multiparameter synchronous convergence principle (MPSC), thereby enhancing the reliability and accuracy of the generated navigation trajectories. In practice, the resolution of geomagnetic sensors imposes a minimum detection distance—typically ranging from a few to tens of kilometers—below which variations in the geomagnetic field cannot be identified [18]. This limitation characterizes geomagnetic navigation as a challenging and expensive optimization problem (EOP) [19], where rapid convergence of the population is crucial to minimize randomness in carrier movement.

Recent efforts to enhance bionic geomagnetic navigation through deep reinforcement learning (DRL) have shown considerable promise in enabling autonomous exploration and promoting the development of more adaptable navigation strategies in complex environments. DRL, a machine learning approach that learns optimal strategies through trial-and-error interactions with the environment, optimizes navigation by leveraging geomagnetic parameters to adapt to dynamic conditions [20]. Wang et al. proposed a geomagnetic navigation algorithm utilizing a deep Q-network (DQN) to determine heading angles based on geomagnetic parameters, achieving superior convergence compared to the evolutionary algorithm [21]. Subsequently, they introduced a deep double Q-network (DDQN) framework, which further decreased the number of steps required to reach targets [22]. However, both methods only considered geomagnetic parameters at the vehicle’s current position, excluding information about the destination. This limitation constrained the models to fixed objectives, thereby reducing their adaptability to changing tasks or environments and necessitating retraining for new scenarios.

### 1.3. Research Significance

Animals demonstrate remarkable magnetotactic behaviors, enabling long-distance migrations to destinations far beyond their typical habitats, despite having no prior knowledge of geomagnetic field distributions [10]. By relying solely on local geomagnetic sensing, they effectively navigate by deducing actions from partial observations. This natural capability underscores the potential for bionic geomagnetic navigation to adopt similar strategies, focusing on learning mechanisms that allow agents to infer optimal actions from limited geomagnetic data. Such an approach could facilitate robust and adaptable navigation in diverse, unmapped environments.

The demonstrated success of DRL in other navigation domains, including visual and robotic navigation [23], highlights its remarkable ability to generalize across environments and solve complex tasks. Unlike conventional methods that rely on predefined models or exhaustive datasets, DRL enables agents to learn adaptable, goal-oriented strategies by extracting high-level patterns from raw sensory inputs. These strategies, designed for resilience and long-term optimization, allow agents to navigate effectively in dynamic and noisy environments without explicit prior knowledge [20,24].

In bionic geomagnetic navigation, DRL’s strengths are particularly relevant. By learning to infer navigation actions from localized geomagnetic data, agents can develop robust strategies adaptable to diverse and uncharted environments. The near-Earth geomagnetic field shows a continuous gradient at a macro scale but is influenced by factors like magnetic rock distribution and tectonic evolution [25], leading to significant spatial heterogeneity. This results in variations in magnetic field strength, gradient direction, and local features across regions. Such regional differences make it difficult for navigation systems to infer global states from local observations, limiting the robustness of traditional algorithms. Therefore, historical observation sequences are needed to establish state associations and develop adaptive strategies for global decision-making, thus generalizing the trained model well in other scenarios.

### 1.4. Article Contributions

In this article, we examine bionic geomagnetic navigation from a partially observable perspective, utilizing temporal dependencies in environmental states to infer geomagnetic field variations that influence navigation decisions. To tackle these challenges, we present a POMDP-based DRL framework that employs belief estimation to enhance decision-making. Our key contributions are as follows:

- We propose a DRL-based bionic geomagnetic navigation framework that does not rely on traditional mapping algorithms. This framework infers optimal actions by sensing the geomagnetic field along the carrier, mimicking the navigation methods of migratory animals that travel long distances without pre-constructed maps.
- We integrate the POMDP to address the challenges of limited geomagnetic observations. The PPO-LSTM algorithm enables the agent to optimize navigation decisions by considering historical observations, ensuring robust performance despite the inherent uncertainty in local geomagnetic sensing.
- We design a dense reward function that provides continuous feedback, ensuring the agent receives informative signals throughout the navigation process. This design combines potential-based and intrinsically motivated rewards to encourage agents to explore actively, resulting in more efficient navigation.
- We construct a long-distance navigation simulation environment and utilize real geomagnetic data from the IGRF-14 geomagnetic model for comprehensive experimental validation. Extensive comparative studies demonstrate our approach’s superior performance over existing heuristic search-based navigation methods, while the effectiveness of our POMDP-based improvements in handling partial observability is thoroughly validated through empirical results.

### 1.5. Article Organization

The rest of the article is organized as follows. Section 2 presents the mathematical description of the geomagnetic navigation from a deep reinforcement learning perspective. Section 3 formally defines the bionic geomagnetic navigation problem through a 7-tuple POMDP formulation, details the proposed DRL framework, and elaborates the dense reward design methodology. Section 4 validates our PPO-LSTM algorithm in ideal and noisy environments through comparative experiments. Finally, Section 5 summarizes findings from the simulation results and discusses future research.

## 2. Fundamentals for Geomagnetic Navigation

### 2.1. Mathematical Description of the Geomagnetic Field

**Figure 1:**
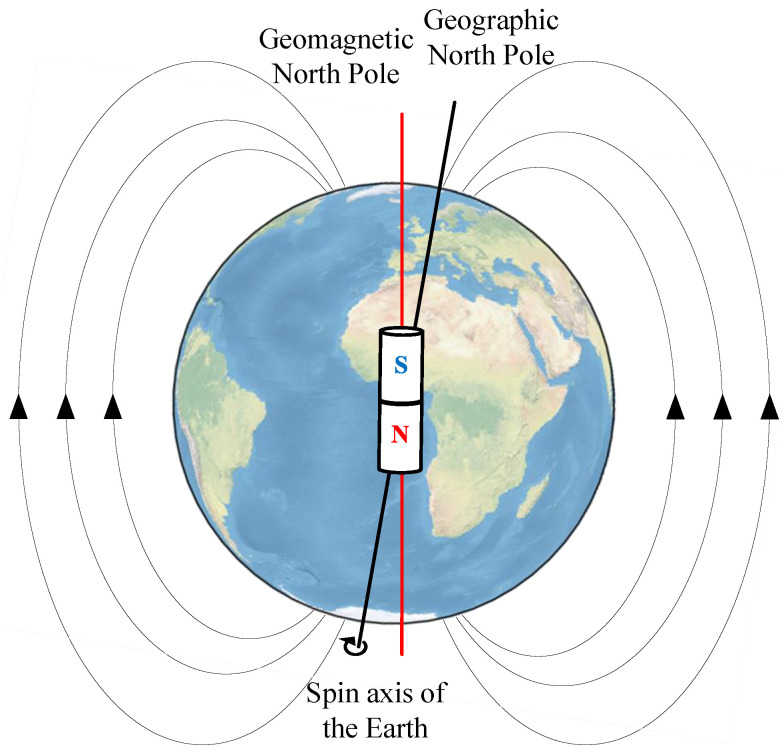
Description of the geomagnetic field and geomagnetic parameters.

The geomagnetic field is a fundamental physical phenomenon of the Earth, generated by the movement of conductive fluids in its core [26]. It remains stable and continuous, exhibiting a surface strength of 30,000 to 60,000 nanoteslas (nT) and slow temporal variations [18]. As shown in Figure 1, the geomagnetic field can be approximated by a dipole model centered at the Earth’s core.

To quantitatively describe the Earth’s magnetic field at any given location, we represent the geomagnetic field as a three-dimensional vector $\vec{B}$ in the local north–east–down (NED) coordinate system:

(1) $$\begin{array}{c} \vec{B}=\left(X,Y,Z\right) \end{array}$$

where *X* is the north component (positive northward), *Y* is the east component (positive eastward), and *Z* is the vertical component (positive downward). This vector encapsulates both the intensity and direction of the geomagnetic field. From $\vec{B}$, we derive the following additional geomagnetic parameters:

- Total Field Intensity (*F*): The overall strength of the magnetic field, calculated as the magnitude of $\vec{B}$:
  (2) $$\begin{array}{c} F=\sqrt{X^{2}+Y^{2}+Z^{2}}. \end{array}$$
- Horizontal Intensity (*H*): The magnitude of the field’s projection onto the horizontal (north–east) plane:
  (3) $$\begin{array}{c} H=\sqrt{X^{2}+Y^{2}}. \end{array}$$
- Declination (*D*): The angle between the horizontal component and true north, computed as follows:
  (4) $$\begin{array}{c} D=\arctan2(Y,X). \end{array}$$
  The arctan2 function considers the signs of both *X* and *Y* to determine the correct quadrant, providing *D* as an angle in the range $\left[-180^{\circ },180^{\circ }\right]$, which resolves the directional ambiguity inherent in traditional methods, like arctan or arccos.
- Inclination (*I*): The angle between the total field vector and the horizontal plane, derived as follows:
  (5) $$\begin{array}{c} I=\arctan\left(\frac{Z}{H}\right). \end{array}$$
  Here, $H=\sqrt{X^{2}+Y^{2}}$ is the magnitude of the horizontal component, ensuring a non-negative denominator. The sign of *Z* determines whether the field tilts downward (I>0) or upward (I<0), with *I* ranging from $-90^{\circ }$ to $90^{\circ }$.

The distribution of geomagnetic parameters across the Earth’s surface varies with latitude and longitude, exhibiting distinct spatial patterns, where *D* primarily varies with longitude, changing gradually near the equator but steeply at high latitudes, especially around the South Atlantic Anomaly and polar regions. *I* transitions from near zero at the geomagnetic equator to ±90° at the poles, with the steepest gradient at mid-latitudes. *F* is strongest near the poles (above 60,000 nT) and weakest in the South Atlantic Anomaly (below 25,000 nT), with sharper gradients at high latitudes. This variation is closely related to *H* and *Z*. *H* reaches its maximum at the geomagnetic equator and decreases toward the poles, whereas *Z* follows the opposite trend, becoming dominant at high latitudes. These variations align with the Earth’s dipole structure, where the horizontal component weakens and the vertical component intensifies as one moves away from the equator. *X* varies significantly near the dip poles and *Y* shows strong longitudinal dependence, particularly in auroral regions [27,28]. These structured variations create a near one-to-one correspondence with geographic locations [29], making the geomagnetic field a reliable reference for long-range navigation.

### 2.2. Bionic Geomagnetic Navigation System Model

We consider a bionic geomagnetic navigation system in which an autonomous agent performs target-driven navigation without relying on external positioning technologies [18]. In this setting, the agent relies solely on local geomagnetic field measurements as a substitute for absolute positioning. In our bio-inspired navigation framework, the agent lacks access to a globally referenced geomagnetic map but knows only the destination’s geomagnetic signature as a reference, iteratively estimating its heading at each time step using real-time geomagnetic observations to progressively approach the destination.

**Figure 2:**
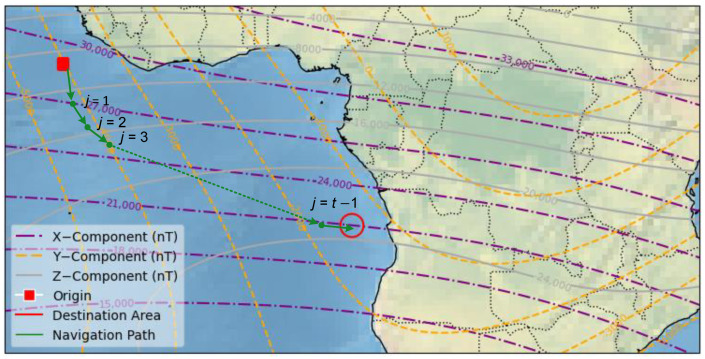
Illustration of the bionic geomagnetic navigation process.

Many navigation scenarios—such as ground and underwater applications—constrain motion to a near-planar surface, where altitude changes are negligible. For simplicity, we model the navigation process in a two-dimensional plane. At each time step *j*, the agent determines a navigation action that updates its position based on its current orientation and the chosen action. This navigation process is illustrated in Figure 2, where the carrier’s trajectory is depicted as it adjusts its heading based on geomagnetic field measurements to reach the destination.

The navigation system continuously assesses whether the terminal condition is satisfied at each time step based on the spatial similarity of the geomagnetic field. Specifically, the Euclidean distance between the measured vector of the geomagnetic parameters $B_{j}=\left(B_{j}^{1},B_{j}^{2},\ldots ,B_{j}^{n}\right)$ and the reference field at the target $B_{t}=\left(B_{t}^{1},B_{t}\ldots ...,B_{t}^{n}\right)$ is computed as follows:

(6) $$\begin{array}{c} d_{j}=\sqrt{\sum_{i=1}^{n}{\left(B_{t}^{i}-B_{j}^{i}\right)}^{2}}. \end{array}$$

where *j* represents the *j*-th measurement of the autonomous agent, corresponding to the *j*-th time step during navigation. The index *i* denotes the *i*-th component of the geomagnetic vector, with *i* ranging from 1 to *n*, where *n* is the number of components of the geomagnetic vector.

To ensure consistent evaluation across different geomagnetic field strengths, we normalize $d_{j}$ by scaling each component’s deviation relative to a reference:

(7) $$\begin{array}{c} d_{j}^{norm}=\sqrt{\sum_{i=1}^{n}{\left(\frac{B_{t}^{i}-B_{j}^{i}}{B_{t}^{i}-B_{0}^{i}}\right)}^{2}}, \end{array}$$

such that this formulation normalizes each component’s deviation $B_{t}^{i}-B_{j}^{i}$ by the reference $B_{t}^{i}-B_{0}^{i}$, ensuring the equitable contribution of geomagnetic parameters with varying ranges in the distance metric. The Euclidean distance form provides a balanced measure of the combined effect of all components.

Theoretically, $d_{j}^{norm}\to 0$ indicates that the autonomous agent has reached the destination [14], as the measured geomagnetic vector perfectly matches the reference vector. However, due to environmental noise and sensor uncertainties, we consider the navigation successful when $d_{j}^{norm}$ falls below a predefined threshold ζ:

(8) $$\begin{array}{c} \lim_{j\to \infty }d_{j}^{norm}\to 0, \end{array}$$

which ensures robust destination recognition while accounting for real-world measurement imperfections.

### 2.3. Partial Observability and Decision Modeling

In real-world navigation, the agent typically measures only the current magnetic field characteristics, lacking direct access to a global geomagnetic map or the ability to predict future environmental changes. Consequently, its decision-making relies on historical magnetic field measurements and movement trajectories, rendering full state observability unattainable. To address this challenge, we model navigation as a POMDP, defined by the tuple $\langle S,A,P_{S},O,P_{O},R,\gamma \rangle$. Here, S and A denote the state and action spaces, respectively, while O represents the observation space. The transition function $P_{S}:S\times A\times S\to \left[0,1\right]$ models the probability of transitioning to the next state $s^{\prime }$ given the current state *s* and action *a*. Unlike fully observable settings, the agent does not have direct access to S but instead receives partial observations drawn from an observation model $P_{O}:S\times A\times O\to \left[0,1\right]$ that specifies the probability of receiving observation *o* given state *s* and action *a*. The reward function R:S×A→R, with γ∈[0,1] as the discount factor.

Belief space B—a probability distribution over possible states—is built from historical observations and actions, helping the agent infer its position and decide moves despite limited information. By incorporating goal-conditioned learning mechanisms, we introduce a goal-augmented POMDP (GA-POMDP) through the integration of a supplementary tuple $\langle G,p_{g},\varphi \rangle$, where G encompasses the target space for navigation objectives, while $p_{g}$ characterizes the underlying distribution of potential destinations in the environment. The mapping function φ:O→G establishes the correspondence between observed states and navigation targets. Our framework aims to derive an optimal policy $\pi_{\theta }:B\times G\times A\to \left[0,1\right]$, parametrized by θ∈Θ, that optimizes the expected cumulative return across the goal distribution:

(9) $$\begin{array}{c} J\left(\pi \right)=E_{a_{j}∼\pi_{\theta }\left(\cdot |b_{j},g\right),g∼p_{g},s_{j^{\prime }}∼P_{S}\left(\cdot |s_{j},a_{j}\right),o_{j}∼P_{O}\left(\cdot |s_{j},a_{j}\right)}\left[\sum_{j}\gamma^{j}r\left(s_{j},a_{j},g\right)\right]. \end{array}$$

## 3. PPO Algorithm Based on POMDP

This section describes the proposed sequential navigation decision based on the PPO framework. First, some key features of this DRL-based framework are reviewed. Next, the state, action, and reward spaces considered for the optimization problem are defined. Finally, the algorithmic solution is derived.

### 3.1. PPO Framework

**Figure 3:**
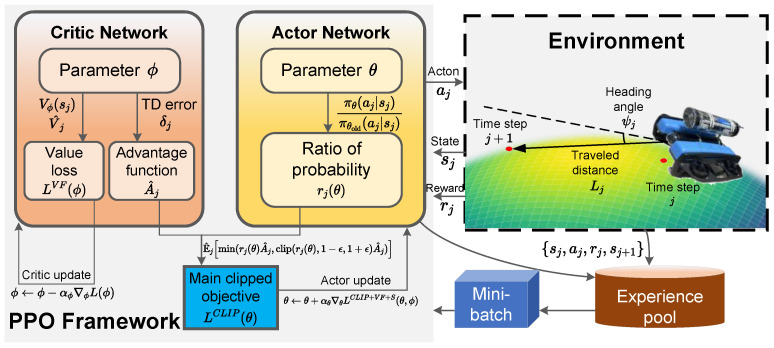
Workflow diagram of PPO algorithm employed.

Proximal policy optimization (PPO) is a widely used DRL algorithm recognized for its balance between training stability and sample efficiency [30]. As an enhancement over earlier policy optimization methods, PPO introduces a clipped surrogate objective, which ensures more reliable and stable updates [31]. The structure of the PPO framework is shown in Figure 3.

PPO optimizes policies through a clipped surrogate objective function that prevents excessive policy updates:

(10) $$L^{CLIP}\left(\theta \right)={\hat{E}}_{j}\left[\min\left(r_{j}\left(\theta \right){\hat{A}}_{j},\mathrm{clip}\left(r_{j}\left(\theta \right),1-\epsilon ,1+\epsilon \right){\hat{A}}_{j}\right)\right],$$

where $r_{j}\left(\theta \right)=\frac{\pi_{\theta }\left(a_{j}|s_{j}\right)}{\pi_{\theta_{old}}\left(a_{j}|s_{j}\right)}$ is the probability ratio between the new and old policies, and ${\hat{A}}_{j}$ is the advantage estimate. The clipping parameter ϵ constrains the ratio to remain within the interval [1−ϵ,1+ϵ], thereby limiting the size of policy updates.

For advantage estimation, PPO commonly employs generalized advantage estimation (GAE):

(11) $${\hat{A}}_{j}=\sum_{l=0}^{\infty }{\left(\gamma \lambda \right)}^{l}\delta_{j+l},$$

where $\delta_{j}=r_{j}+\gamma V\left(s_{j+1}\right)-V\left(s_{j}\right)$ is the temporal difference error, γ is the discount factor, and λ is the GAE parameter that controls the bias–variance tradeoff.

The main features of the PPO framework that make it a suitable choice for the bionic geomagnetic navigation problem are as follows:

- Unlike value-based methods such as a DQN, which struggle with continuous action spaces [21,22], PPO directly optimizes policies, making it particularly effective for high-dimensional control tasks. Its actor–critic architecture allows for the efficient mapping of sensory inputs to actions. In bionic geomagnetic navigation, where agents adjust their trajectories based on variations in the magnetic field, PPO facilitates smooth and adaptive control, outperforming approaches that rely on discrete actions.
- PPO learns a stochastic policy by sampling actions from a probability distribution instead of selecting them deterministically [32], enhancing exploration and helping prevent premature convergence to suboptimal strategies. Given the uncertainties of environmental variability in geomagnetic navigation, a stochastic policy enhances adaptability and generalization across various scenarios. The policy gradient updates of PPO ensure efficient exploration without introducing excessive randomness.
- As an on-policy algorithm, PPO updates its policy based on recent interactions rather than relying on stored experiences. Although on-policy learning is generally less sample-efficient than off-policy methods such as soft actor–critic (SAC), it offers more reliable performance. For geomagnetic navigation, where real-time policy refinement is essential, the PPO’s clipped surrogate objective prevents drastic updates, thereby enhancing training stability and making it suitable for real-world applications.

### 3.2. State, Action and Reward

To address the bionic geomagnetic navigation problem outlined in Equation (9), we define the state and action, among other elements. The elements within our proposed framework are defined as follows:

#### 3.2.1. State Space

Consider a mobile carrier tasked with navigating a large-scale environment using only local magnetic field data and leveraging the spatial variation in the geomagnetic field to guide the carrier toward a destination. The agent does not require knowledge of the carrier’s absolute position; instead, it adjusts its heading based on trends in magnetic field observations to converge toward a predefined target value. The state space must encapsulate the agent’s directional information and historical context; thus, we define the state s∈S as a tuple:

(12) $$\begin{array}{c} s=(\phi ,h_{\mathrm{traj}},h_{\mathrm{mag}}), \end{array}$$

where ϕ∈[−π,π) is the carrier’s orientation relative to a reference direction (e.g., magnetic north); $h_{\mathrm{traj}}\in R^{m\times 2}$ is the sequence of actions over the past *m* time steps, with each entry $\left(\psi_{j-i},L_{j-i}\right)$ for i=1,…,m representing the heading angle and traveled distance at time j−i, providing context for the agent’s path; and $h_{\mathrm{mag}}\in R^{m\times 3}$ is the record of the magnetic field measurements over the past *m* time steps, with each entry $\left[X_{j-i},Y_{j-i},Z_{j-i}\right]$ denoting the three-axis magnetic field components at time j−i, capturing temporal trends in the geomagnetic field.

In this formulation, the orientation ϕ acts as the primary control variable, while $h_{\mathrm{traj}}$ and $h_{\mathrm{mag}}$ supply historical data to infer movement patterns and field gradients. This state-space design leverages these elements to ensure robustness in environments where field-to-location correlations are unavailable, facilitating effective long-distance navigation.

#### 3.2.2. Observation Function and Observation Space

Within a POMDP, the observation space comprises the information directly accessible to the agent at each time step. For an agent navigating via magnetic field data, this space must reflect its sensory capabilities, forming a subset of the state space. We define the observation o∈O as follows:

(13) $$\begin{array}{c} o=[X_{j},Y_{j},Z_{j},X_{t},Y_{t},Z_{t}], \end{array}$$

where each entry $\left[X_{j},Y_{j},Z_{j}\right]\in R^{3}$ represents the three-axis magnetic field component measurement at current time *j*, obtained directly from the magnetometer, reflecting the instantaneous geomagnetic field, and $\left[X_{t},Y_{t},Z_{t}\right]\in R^{3}$ is the current magnetic field component of the destination. Compared to a non-goal-augmented POMDP, where observations lack explicit goal-related information, this approach improves adaptability to specific task requirements by embedding the navigation objective directly into the observation space.

#### 3.2.3. Action Space

Action a∈A is defined as a tuple, a=(ψ,L), where ψ∈[−π,π) is the heading angle, determining the change in orientation; traveled distance $L\in R^{+}$ is the step size, representing the distance moved in the direction of the updated orientation. The heading angle ψ plays a pivotal role in this navigation framework by adjusting the agent’s direction based on inferred trends in the geomagnetic field, directly influencing its trajectory. Complementing this, the step size *L* is constrained to a range of 3 to 5 km. This limitation arises from the precision of geomagnetic sensors [18], which can reliably detect distinct variations in geomagnetic field parameters only over distances of several kilometers. By setting *L* within this range, each movement ensures meaningful changes in the observed magnetic field, thereby facilitating accurate navigation decisions.

#### 3.2.4. Transition Function

The transition function in our POMDP model governs how the agent’s state evolves based on its navigation actions within the bionic geomagnetic navigation system. At each time step *j*, the agent selects a navigation action $a_{j}=\left(\psi_{j+1},L_{j+1}\right)$, driving the state transition from $s_{j}$ to $s_{j+1}=\left(\phi_{j+1},h_{\mathrm{traj},j+1},h_{\mathrm{mag},j+1}\right)$.

The state components update as follows: the orientation is adjusted based on the heading angle, the trajectory history is extended by appending the new action, and the geomagnetic field history incorporates the new observation after the action. Specifically, the orientation update is given by the following:

(14) $$\begin{array}{c} \phi_{j+1}=\phi_{j}+\psi_{j+1}, \end{array}$$

which reflects the direct influence of the action’s heading adjustment on the agent’s direction. The trajectory history $h_{\mathrm{traj},j}$ is updated by appending the action $a_{j}$:

(15) $$\begin{array}{c} h_{\mathrm{traj},j+1}=h_{\mathrm{traj},j}⊕a_{j}, \end{array}$$

where ⊕ denotes concatenation, effectively recording the sequence of navigation decisions.

For the geomagnetic field history, the update involves incorporating the new geomagnetic observation $B_{j+1}$, yielding $h_{\mathrm{mag},j+1}=h_{\mathrm{mag},j}⊕B_{j+1}$. Here, $B_{j+1}$ is the measurement at the agent’s new position after the action, where the position updates kinematically as $x_{j+1}=x_{j}+L_{j+1}\cos\left(\phi_{j}+\psi_{j+1}\right)$ and $y_{j+1}=y_{j}+L_{j+1}\sin\left(\phi_{j}+\psi_{j+1}\right)$. Assuming a complex mapping between the position and geomagnetic field, $B_{j+1}=f\left(x_{j+1},y_{j+1}\right)$, this relationship can be expressed as follows:

(16) $$\begin{array}{c} B_{j+1}=f\left(x_{j}+L_{j+1}\cos\left(\phi_{j}+\psi_{j+1}\right),y_{j}+L_{j+1}\sin\left(\phi_{j}+\psi_{j+1}\right)\right), \end{array}$$

allowing the geomagnetic observation update to reflect the carrier’s movement and maintain consistency with the state space through interaction with the environment.

#### 3.2.5. Reward Function

The reward function R:S×A×S→R is designed to guide the agent toward the target in our bionic geomagnetic navigation system by leveraging real-time geomagnetic field observations, capitalizing on the Earth’s relatively uniform magnetic field distribution to provide consistent navigation cues. Initial experiments with a simple reward, where the agent earns a substantial positive reward $r_{\mathrm{goal}}$ only when it reaches the target—defined as when the geomagnetic distance $d_{j}<\zeta$—and receives zero otherwise. However, the long-horizon nature of navigation tasks makes it hard for the agent to connect distant rewards to current actions, resulting in inefficient exploitation and slow learning. To give fine-grained feedback signals, we developed a three-part reward function that blends sparse and dense elements; the updated reward function is expressed as follows:

(17) $$\begin{array}{c} R\left(j\right)=\left\{\begin{array}{cc} r_{\mathrm{goal}}, & \mathrm{if}\;d_{j}<\zeta \\ \alpha (d_{j}-d_{j-1})+c, & \mathrm{otherwise} \end{array}\right., \end{array}$$

where $r_{\mathrm{goal}}$ is a large positive reward awarded when the agent reaches the target, $\alpha (d_{j-1}-d_{j})$ provides dense feedback based on progress toward the target with α>0 as a scaling factor, and *c* serves as an intrinsic reward to guide the agent’s heading. The term $d_{j}$ represents the geomagnetic distance, calculated as follows:

(18) $$\begin{array}{c} d_{j}=\sqrt{{\left(X_{j}-X_{t}\right)}^{2}+{\left(Y_{j}-Y_{t}\right)}^{2}+{\left(Z_{j}-Z_{t}\right)}^{2}}, \end{array}$$

where $B_{j}=\left[X_{j},Y_{j},Z_{j}\right]$ is the geomagnetic field vector observed at the agent’s current position at step *j*, and $B_{t}=\left[X_{t},Y_{t},Z_{t}\right]$ is the target’s known geomagnetic signature. The condition $d_{j}<\zeta$ defines the target proximity threshold, with ζ as a small positive constant.

*c* is a small reward, and under the assumption of a globally uniform geomagnetic field distribution, *c* leverages the principle of multiparameter simultaneous convergence. This principle predicts a heading angle, $\phi_{j}^{\prime }$, which enables multiple geomagnetic parameters to converge toward the target’s values at consistent rates [13]. Although this predicted heading angle $\phi_{j}^{\prime }$ may exhibit deviations due to local field variations, it remains a valuable directional cue for the agent. The specific formulation of *c* is as follows:

(19) $$\begin{array}{c} c=\beta \left(\frac{\pi }{4}-\left|\phi_{j}-\phi_{j}^{\prime }\right|\right), \end{array}$$

where β>0 is a scaling coefficient. If the heading is close (less than $\frac{\pi }{4}$), *c* is positive; if not, it is negative. This helps the agent move better using the field’s steady spread.

### 3.3. The PPO-LSTM Algorithm

**Figure 4:**
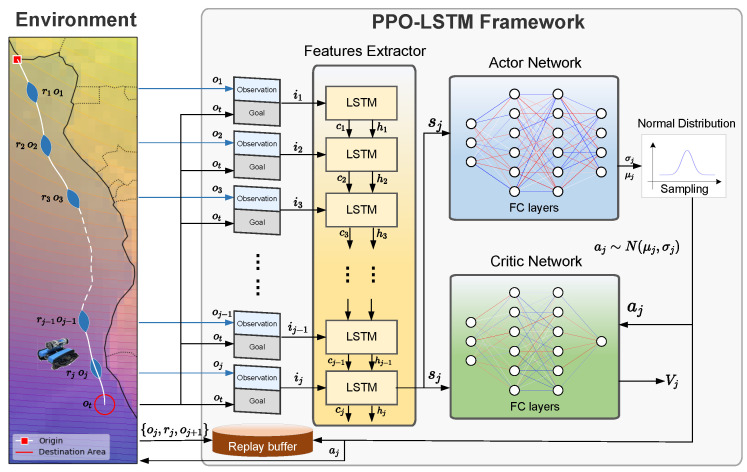
PPO-LSTM framework for bionic geomagnetic navigation, showing the integration of LSTM cells within the actor–critic architecture to process sequential observations and goals.

Building on the PPO framework discussed previously, we now introduce a recurrent neural network extension that addresses the temporal dependencies inherent in geomagnetic navigation. The proposed PPO-LSTM framework for bionic geomagnetic navigation is illustrated in Figure 4. The architecture integrates LSTM cells into the standard PPO algorithm to process sequential observations and capture temporal dependencies, effectively transforming the POMDP into a more manageable MDP.

As shown in Figure 4, the PPO-LSTM architecture consists of several key components: an integrated feature extraction and state representation module, which processes the concatenated sensory observation and goal through LSTM networks, where the hidden states $h_{j}$ serve as representations of the underlying POMDP state $s_{j}$, while the cell states $c_{j}$ reflect long-term dependencies; actor and critic networks that utilize the LSTM’s hidden states to incorporate temporal context and make decisions; and a replay buffer that stores experiences for training.

The PPO-LSTM algorithm extends standard PPO by conditioning both the policy and value function on the LSTM hidden state, which serves as an estimate of the true environment state:

(20) $$\pi_{\theta }\left(a_{j}|h_{j}\right)\;\mathrm{and}\;V_{\phi }\left(h_{j}\right),$$

where $h_{j}$ is the hidden state of the LSTM at time *j*, representing our best estimate of the underlying state $s_{j}$ in the POMDP formulation. This conditioning enables the agent to make decisions based not just on current observations but on a more complete representation of the environment state encoded in the LSTM hidden state. The complete PPO-LSTM algorithm for bionic geomagnetic navigation is presented in Algorithm 1.

Due to the integration of a sequential network like LSTM, the PPO-LSTM algorithm requires specific adaptations to perform effectively. It is essential to process sequential data accurately to preserve the integrity of the LSTM states, ensuring that the network retains patterns from historical data—a critical aspect for our model to accurately capture temporal dependencies.

The incorporation of LSTM into the PPO framework is particularly beneficial for addressing the partial observability of the geomagnetic navigation problem as formulated in our POMDP model. By using the LSTM hidden state $h_{j}$ as a representation of the underlying state $s_{j}$, we effectively transform the POMDP into an MDP from the agent’s perspective. The cell state $c_{j}$, although not directly fed into the actor and critic networks, indirectly participates in the decision-making process by influencing the computation of $h_{j}$ at each time step. The hidden state $h_{j}$ can encode information from the history of observations, allowing the agent to disambiguate perceptually similar states, and the cell state $c_{j}$ preserves long-term information about the environment, enabling adaptation to slowly changing conditions even when this information is not immediately reflected in $h_{j}$.
**Algorithm 1:** PPO-LSTM for Bionic Geomagnetic Navigation
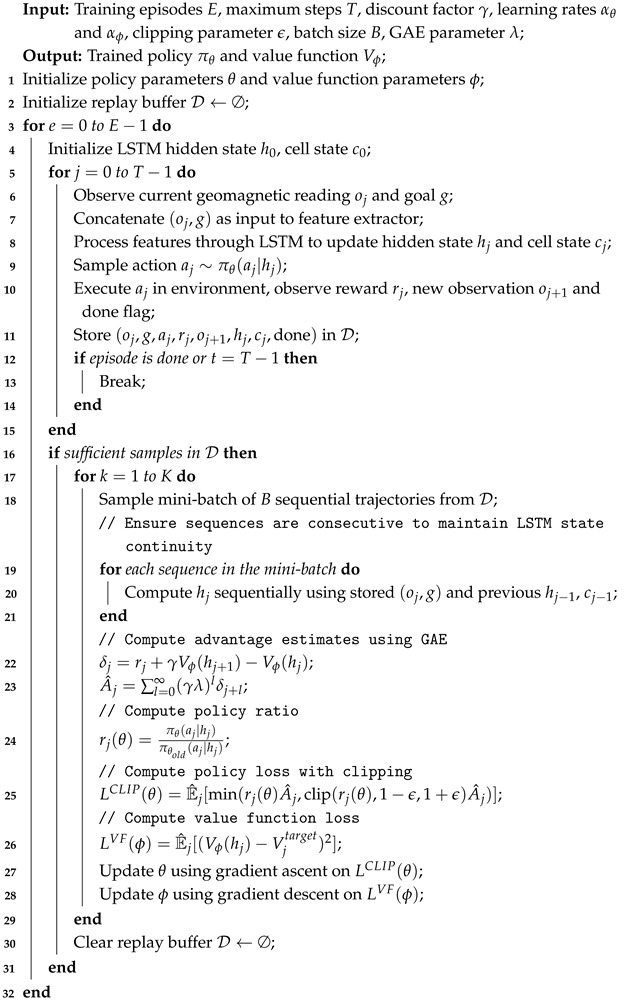


## 4. Algorithm Implementation and Result

To evaluate the performance of the proposed PPO-LSTM algorithm for bionic geomagnetic navigation, we conducted a series of experiments on a laptop equipped with the following specifications: a 12th Gen Intel® Core™ i7-12700 processor (20 cores, manufactured by Intel Corporation, Santa Clara, CA, USA), 62.6 GiB of RAM, and running Ubuntu 20.04.6 LTS. Given that our task does not involve high-dimensional data, we opted not to employ GPU acceleration. This decision facilitates efficient training and evaluation on a CPU-based system, leveraging the ample processing power and memory available.

### 4.1. Experimental Setup

**Figure 5:**
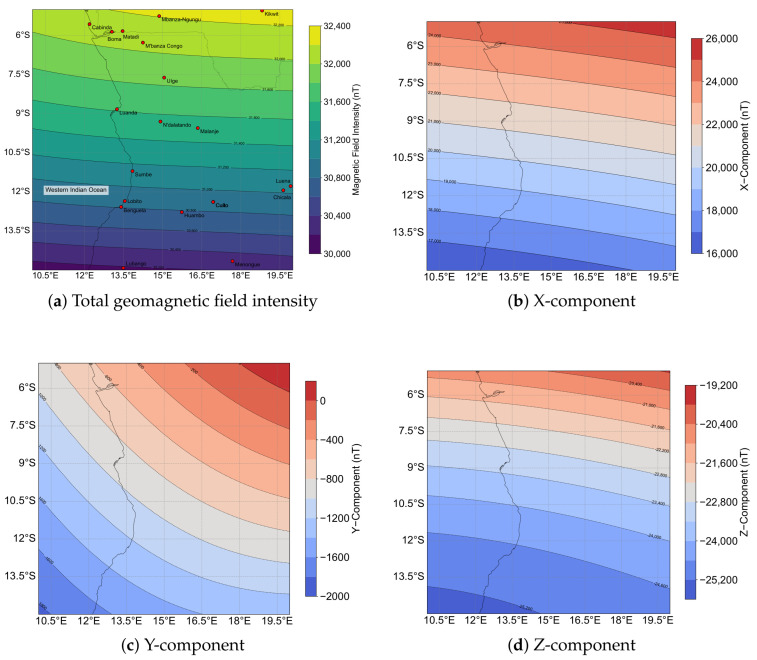
Geomagnetic field distribution over the simulation region (5° S–15° S, 10° E–20° E). (**a**) Total field intensity derived from the IGRF-14 geomagnetic model (EPSG:32333), with key cities and geographical features annotated. (**b**) Spatial distribution of the X-component. (**c**) Spatial distribution of the Y-component. (**d**) Spatial distribution of the Z-component.

We selected a geographical region spanning latitudes from 5° S to 15° S and longitudes from 10° E to 20° E, covering a 10° by 10° area. Situated over the southern part of South Sudan and the western Indian Ocean, this region is near the equator and within the influence of the South Atlantic Anomaly. The South Atlantic Anomaly is known for its unique geomagnetic characteristics, including lower magnetic field intensity and higher variability [33], which introduces distinct challenges for geomagnetic navigation. This choice facilitates realistic geomagnetic simulations, ensuring reliable evaluation of the algorithm’s performance. The geomagnetic field distribution across this region is illustrated in Figure 5, where subplot (a) presents the total field intensity, highlighting key geographical features and cities, while subplots (b)–(d) depict the X, Y, and Z components, respectively. These figures illustrate the distinct spatial patterns of each component across the region, thereby demonstrating the spatial variability in the magnetic field, which is critical for testing navigation accuracy in our simulation.

We developed the simulation environment for bionic geomagnetic navigation using the Gymnasium library [34], a robust framework for creating and testing reinforcement learning scenarios. Within this environment, we randomly designated navigation starting and ending points across the defined region. The magnetic field data for our simulation area is sourced from the IGRF-14 geomagnetic model [35], which offers an accurate and up-to-date representation of the Earth’s magnetic field. To ensure seamless integration of this data into our simulation, we performed coordinate transformations using the PROJ library with the EPSG:32333 projection, aligning the magnetic data precisely with our chosen region.

The PPO-LSTM algorithm, designed to optimize navigation decisions, determines the optimal heading angle and traveled distance at each step of the navigation process. To achieve stable and effective training, the hyperparameters for PPO-LSTM were tuned using Optuna, an open-source framework that employs Bayesian optimization to efficiently explore the hyperparameter space. The hyperparameters optimized by Optuna are presented in Table 1.

### 4.2. Success Rate and Reward Convergence

**Figure 6:**
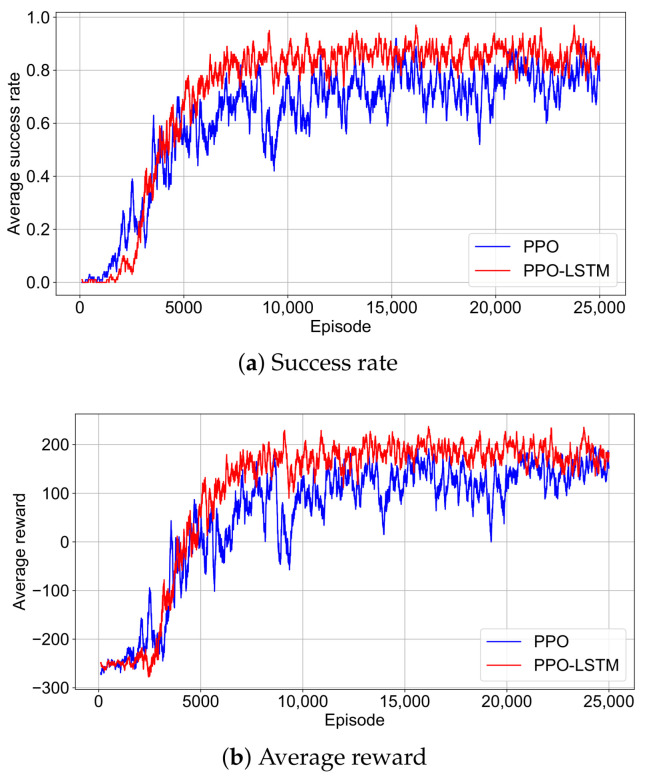
Smoothed success rate and reward trajectories during training for PPO and PPO-LSTM. (**a**) Success rate over training episodes. (**b**) Average reward over training episodes.

To investigate the performance of our POMDP-based approach in the bionic geomagnetic navigation task, we examined the training dynamics of the PPO and PPO-LSTM. Here, we analyze these dynamics through the success rate and reward trajectories over 25,000 episodes as shown in Figure 6. The success rate is defined as the proportion of episodes in which the agent reaches the destination within the maximum allowed steps. To mitigate the noise inherent in reinforcement learning, we apply a sliding window average with a length of 100 episodes to smooth both the success rate and reward data across episodes.

The success rate of PPO increases more gradually and exhibits noticeable oscillations, indicating less consistent progress without temporal memory. Its reward curve similarly shows a slower, fluctuating rise, only approaching PPO-LSTM’s level after 20,000 episodes, with greater variability even late in training. This suggests that PPO’s policy optimization is less efficient and more unstable compared to PPO-LSTM’s smoother convergence.

Conversely, PPO-LSTM exhibits a stable and steep initial rise in both success rate and average reward, surpassing PPO’s success rate within 5000 episodes. This rapid ascent demonstrates efficient policy optimization, driven by the LSTM’s ability to leverage past geomagnetic observations. For practical autonomous navigation systems, PPO-LSTM’s quick convergence to high success rates and stable rewards reduces training costs and ensures dependable performance.

### 4.3. Imitation in Long-Range Navigation Missions Between Cities

**Figure 7:**
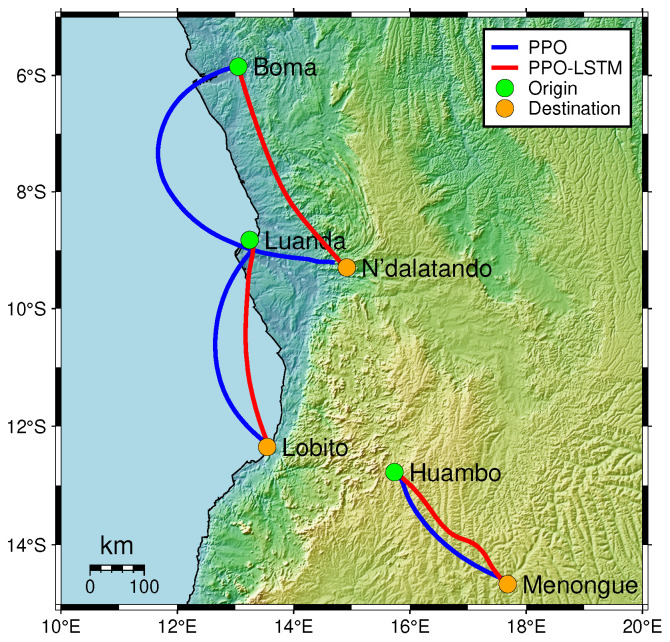
Navigation trajectories in long-range travel tasks under ideal conditions, utilizing GMT Earth relief and natural Earth data. Red and blue thick solid lines represent PPO-LSTM and PPO trajectories, respectively.

To evaluate the effectiveness of our POMDP-based approach in long-range geomagnetic navigation, we conducted experiments under ideal conditions, devoid of sensor noise. We designed three navigation tasks between pairs of cities within a selected region: Boma to N’dalatando, Luanda to Lobito, and Huambo to Menongue. These tasks involve straight-line distances ranging from approximately 300 to 500 km, specifically chosen to test the algorithm’s capability over extended ranges. The city pairs were selected to represent diverse geographical contexts—spanning coastal to inland areas and crossing national borders—introducing varied geomagnetic field characteristics and spatial orientations. This experimental design serves to validate the fundamental effectiveness of our approach, providing a baseline assessment of its adaptability and precision in long-range navigation tasks. We utilized the model that exhibited the best performance during the training phase. The navigation trajectories generated for these tasks are presented in Figure 7, which visually depicts the paths taken by the agent under different algorithmic models.

The trajectories illustrated in Figure 7 reveal a clear performance distinction between the standard PPO and the PPO-LSTM models across all three navigation tasks. The PPO-LSTM model consistently produced more direct paths compared to the standard PPO. For example, in the Luanda to Lobito task, PPO-LSTM maintained a nearly straight trajectory, while PPO showed noticeable deviations. Similarly, in the Boma to N’dalatando task, PPO-LSTM navigated with greater stability, whereas PPO exhibited larger detours. Even in the shorter task from Huambo to Menongue, PPO-LSTM exhibited oscillations but still outperformed PPO.

A common characteristic observed in the PPO trajectories is their tendency to exhibit a distinct curvature, rather than following a straight-line path to the target. This arcshaped behavior is particularly evident across all three tasks and warrants further analysis. The curvature in PPO trajectories may be linked to the standard PPO model’s limited ability to adapt to rapid changes in the geomagnetic field’s components. Standard PPO, lacking temporal memory, struggles to account for these gradual shifts, leading to undercorrections in its heading. In contrast, PPO-LSTM leverages its memory component to maintain a historical record of geomagnetic observations. This temporal awareness allows it to anticipate and smooth out responses to spatial gradients in the field.

### 4.4. Navigation Trajectory with Sensor Noise

In practical applications, geomagnetic measurements are inherently subject to sensor noise, which can significantly influence the performance of navigation algorithms. Building upon the idealized, noise-free experimental conditions discussed earlier, this subsection extends the analysis by incorporating the effects of sensor noise on navigation trajectories. To ensure the realism and relevance of our simulations, we first establish the rationality of introducing sensor noise by characterizing the noise properties of a real-world magnetometer.

**Figure 8:**
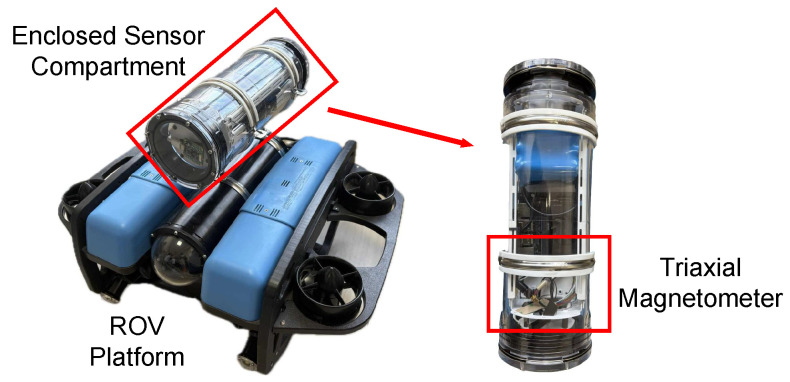
Experimental setup of the ROV platform with the RM3100 triaxial magnetometer for magnetic field data collection in a static environment.

**Figure 9:**
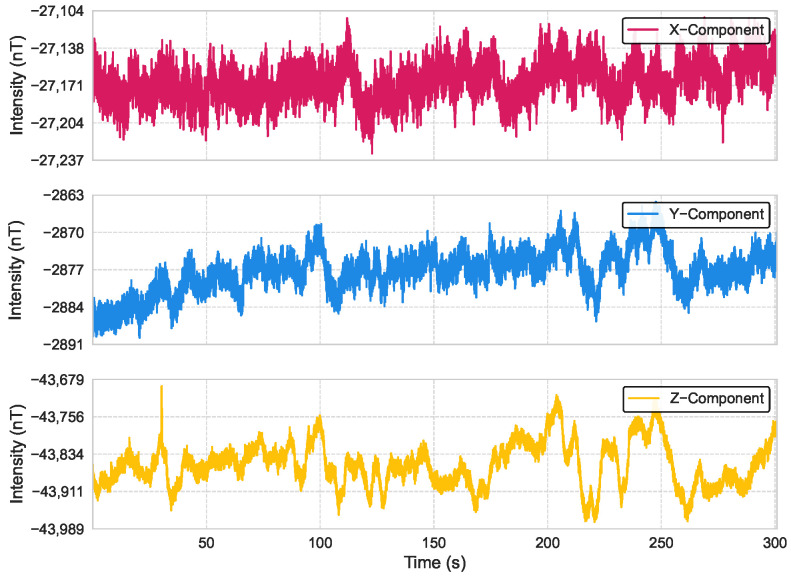
Time-domain waveforms of three-axis magnetic field intensities measured by the RM3100 magnetometer over a 300 s period at a 100 Hz sampling rate.

We conducted an experiment in an open, interference-free area where a remotely operated vehicle (ROV) platform, equipped with an RM3100 triaxial magnetometer (manufactured by PNI Sensor Corporation, Santa Rosa, CA, USA) and an NVIDIA Jetson device (manufactured by NVIDIA Corporation, Santa Clara, CA, USA) for onboard processing, was kept stationary. The measurement platform and apparatus are illustrated in Figure 8. Magnetic field data were collected at a sampling rate of 100 Hz over a duration of 300 s. The time-domain waveforms of the three-axis magnetic field intensities over the 300 s period are presented in Figure 9; the analysis of the collected data revealed that the noise approximates a Gaussian distribution, with axis-specific standard deviations of $\sigma_{x}=44.3\;\mathrm{nT}$, $\sigma_{y}=62.8\;\mathrm{nT}$, and $\sigma_{z}=117.2\;\mathrm{nT}$. These values, derived from the empirical measurements, serve as baseline parameters for the noise model in our simulations.

As specified by the manufacturer, the RM3100 magnetometer’s theoretical noise include a noise floor of 15–30 nT, temperature variations may introduce drifts of 20–50 nT, and electronic noise from power supplies and circuits can contribute random fluctuations of 10–20 nT. In real-world navigation scenarios, such as long-distance operations in underwater or aerial environments, interference is often less pronounced than in our stationary setup.

We injected Gaussian noise with a standard deviation of 100 nT into each axis. While this value exceeds the typical noise levels of high-precision magnetometers, a measurement sequence we obtained as shown in Figure 9 revealed significant external interference, with noise levels approaching 50–100 nT due to factors such as nearby vehicles and power lines. Thus, adopting a higher noise level of 100 nT in our simulations ensures the algorithm’s robustness in challenging environments with substantial interference, enhancing its reliability for practical deployment in diverse scenarios. To evaluate the impact of this noise on navigation performance, we randomly selected the starting and ending points of navigation tasks within a designated area, with a total of 300 test cases evaluated.

In Table 2, we report the success rate (SR) and success weighted by the path length (SPL) of the PPO and the PPO-LSTM before and after noise introduction. The SR quantifies the agent’s ability to reach the goal, calculated as $\mathrm{SR}=\frac{1}{K}\sum_{i=1}^{K}{\mathrm{Succ}}_{i}$, where K=300 is the total number of tasks, and ${\mathrm{Succ}}_{i}$ is a binary indicator of success (1 if successful, 0 otherwise) for the *i*-th task. The SPL measures the agent’s efficiency in successful tasks, defined as $\mathrm{SPL}=\frac{1}{K}\sum_{i=1}^{K}{\mathrm{Succ}}_{i}\left(\frac{L_{i}^{*}}{\max(L_{i},L_{i}^{*})}\right)$, where $L_{i}$ is the actual path length traveled by the vehicle, and $L_{i}^{*}$ is the straight-line distance between the starting and ending points.

In noise-free conditions, PPO achieves an SR of 94.7%, with 284 successful runs out of 300 experiments, and an SPL of 74.41856%, while PPO-LSTM slightly outperforms it with an SR of 95.7% (287 successes out of 300) and an SPL of 84.89380%, indicating better path efficiency. Under noisy conditions, PPO’s SR drops to 76.3% (229 successful runs) and SPL to 57.92835%, whereas PPO-LSTM maintains higher values at 79.3% (238 successes) and 65.88631%, demonstrating greater robustness to noise through effective noise filtering and decision stabilization.

**Figure 10:**
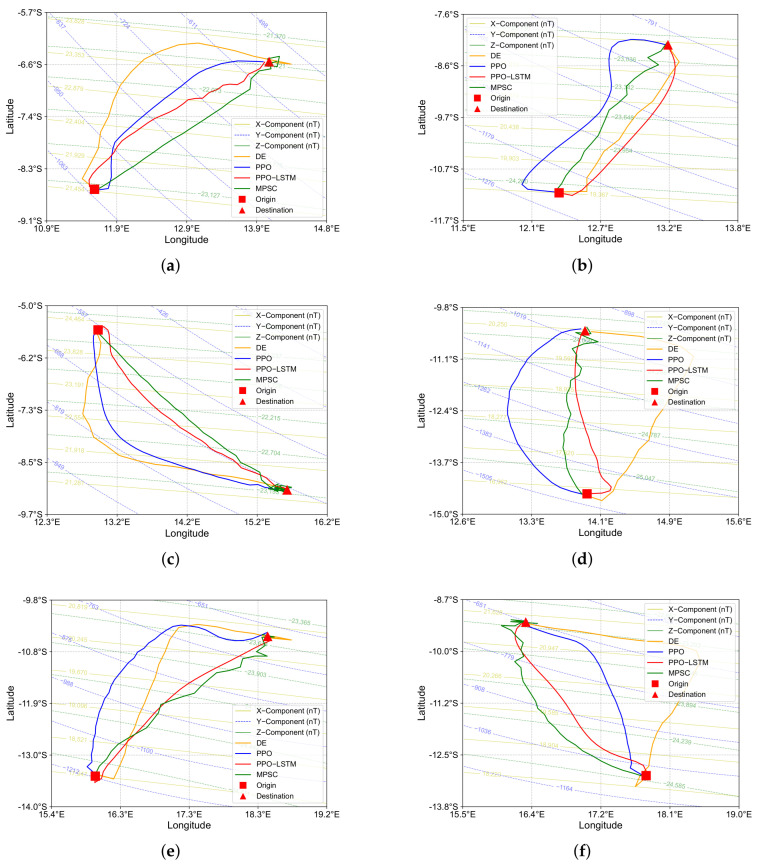
Trajectories of six representative navigation tasks under noisy conditions, comparing PPO, PPO-LSTM, MPSC, and DE algorithms. (**a**) First task trajectory. (**b**) Second task trajectory. (**c**) Third task trajectory. (**d**) Fourth task trajectory. (**e**) Fifth task trajectory. (**f**) Sixth task trajectory. The orange, blue, red, and green thick solid lines represent DE, PPO, PPO-LSTM, and MPSC trajectories, respectively.

We analyze the trajectories of six tasks under noisy conditions in Figure 10, comparing PPO, PPO-LSTM, MPSC [18], and DE [36]. The learning-based algorithms, PPO and PPO-LSTM, produce significantly smoother trajectories than MPSC and DE, with PPO-LSTM achieving more direct paths. In contrast, DE exhibits oscillatory trajectories with frequent detours due to its random search mechanism under noise, while MPSC, despite generating relatively straight paths in simpler scenarios, struggles with deviations in regions with curved geomagnetic contours, leading to suboptimal convergence near the target.

We compute the mean squared error (MSE) and mean absolute error (MAE) for two critical metrics—trajectory smoothness and heading deviation—across six representative tasks. These metrics provide a numerical foundation for evaluating how effectively each algorithm manages noise and sustains navigation performance.

Trajectory smoothness quantifies the consistency of direction changes along the path, with lower values indicating a smoother trajectory. For a trajectory with *N* segments, let $\theta_{i}$ be the direction angle of the *i*-th segment, $\Delta \theta_{i}=\theta_{i+1}-\theta_{i}$, and the second-order difference $\Delta^{2}\theta_{i}=\Delta \theta_{i+1}-\Delta \theta_{i}$. ${\mathrm{MSE}}_{\mathrm{smoothness}}=\frac{1}{N-2}\sum_{i=1}^{N-2}{\left(\Delta^{2}\theta_{i}\right)}^{2}$, and ${\mathrm{MAE}}_{\mathrm{smoothness}}=\frac{1}{N-2}\sum_{i=1}^{N-2}\left|\Delta^{2}\theta_{i}\right|$. Heading deviation measures the discrepancy between the agent’s actual heading and the ideal heading toward the target, with smaller values indicating better alignment. For each point *i*, let $\phi_{i}$ be the actual heading, $\psi_{i}$ the ideal heading, and $\delta_{i}=\phi_{i}-\psi_{i}$ (normalized to [−π,π]). ${\mathrm{MSE}}_{\mathrm{heading}}=\frac{1}{N-1}\sum_{i=1}^{N-1}\delta_{i}^{2}$, and ${\mathrm{MAE}}_{\mathrm{heading}}=\frac{1}{N-1}\sum_{i=1}^{N-1}\left|\delta_{i}\right|$.

The MSE and MAE for both metrics are presented for each algorithm across all six tasks in Table 3. PPO-LSTM consistently outperforms the others, achieving the lowest average MSE and MAE for both smoothness (MSE: 0.0625; MAE: 0.1243) and heading deviation (MSE: 0.0657; MAE: 0.2066). PPO follows with moderate performance, evidenced by slightly higher smoothness errors (MSE: 0.0760; MAE: 0.1499) and significantly elevated heading deviation errors (MSE: 0.2641; MAE: 0.4297), indicating a reduced capacity to maintain target alignment under noise compared to PPO-LSTM.

As two established approaches in bionic geomagnetic navigation, MPSC and DE contrast with the novel reinforcement learning framework introduced by PPO-LSTM. MPSC exhibits markedly higher heading deviation. Theoretically, MPSC demonstrates higher efficiency when the geomagnetic field’s contour lines exhibit high linearity, as its gradient-based approach aligns effectively with straight-line gradients. However, in regions where the contour lines are curved, MPSC’s fixed step length and gradient-based mechanism lacks the adaptability of reinforcement learning algorithms like PPO-LSTM and PPO, hampering its ability to refine navigation near the target and often leading to overshooting or oscillatory behavior around the goal, as observed in Tasks 3 and 6.

DE, with an average smoothness MSE of 1.1364 and MAE of 0.4916, and the highest average heading deviation errors (MSE: 0.6086; MAE: 0.5498), also faces challenges. Its reliance on random search, without a mechanism for learning from past iterations, results in trajectories that are less smooth and stable compared to reinforcement learning-based methods, which benefit from policy optimization over time. This limitation is particularly evident in Task 5, where DE’s heading deviation MSE reaches 1.0579.

These observations lead to the conclusion that PPO-LSTM’s advantages stem from its ability to learn and adapt through reinforcement learning and temporal modeling, enabling it to produce smoother and more direct paths even in noisy environments. In contrast, MPSC’s reliance on fixed gradient-based rules and DE’s dependence on random search without learning capabilities restrict their robustness in complex, noisy conditions.

**Figure 11:**
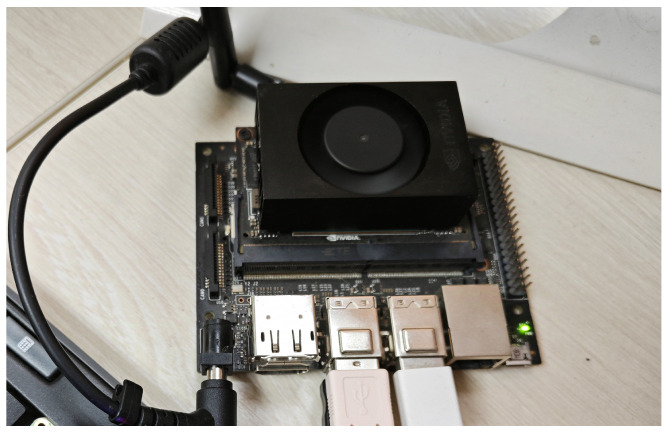
NVIDIA Jetson Nano running the navigation program.

**Figure 12:**
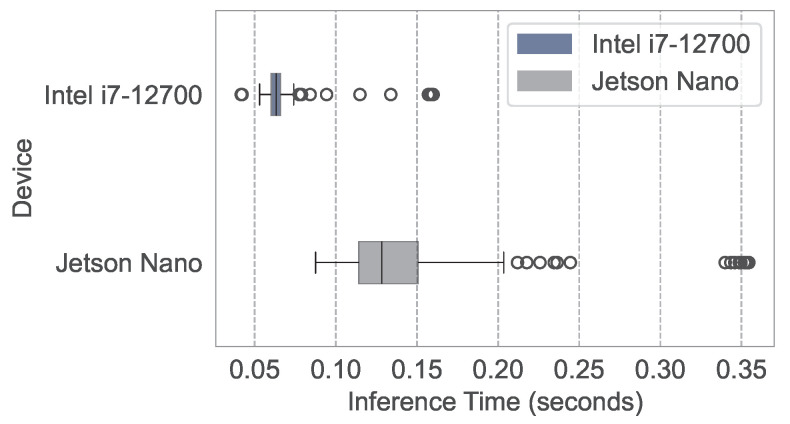
Distribution of PPO-LSTM computation times on Intel i7-12700 and Jetson Nano.

To evaluate the practical feasibility of the PPO-LSTM algorithm, we assessed its computational performance by implementing it on two distinct platforms: a high-performance laptop equipped with an Intel i7-12700 processor and an NVIDIA Jetson device. The Jetson, previously integrated into the ROV platform as shown in Figure 8, was removed for this experiment to independently verify the proposed navigation algorithm’s efficiency on resource-constrained edge devices, with the standalone setup illustrated in Figure 11. The box plot, presented in Figure 12, serves as a graphical representation of the computation times across these devices. The data was collected by executing the algorithm over 200 identical navigation tasks on each platform.

For the laptop, the median computation time is 0.06 s, with an IQR spanning 0.06 to 0.07 s and a full range of 0.04 to 0.16 s. In contrast, the Jetson device records a median computation time of 0.13 s, with an IQR from 0.11 to 0.15 s and a range of 0.09 to 0.35 s. The laptop outperforms the Jetson device by approximately 2 times in terms of median computation time.

Despite the slower performance on the Jetson device, its ability to maintain acceptable computation times for real-time applications confirms that PPO-LSTM is well suited for deployment on resource-constrained edge devices like the Jetson.

## 5. Conclusions

In this paper, the geomagnetic navigation problem was then formulated as a POMDP, and we employed PPO-LSTM to address the challenges of partial observability and sensor noise. Extensive experiments using real-world geomagnetic data from the IGRF-14 geomagnetic model demonstrated that PPO-LSTM outperforms baseline algorithms. The success of PPO-LSTM highlights the critical role of temporal modeling in navigation tasks, and the LSTM component enables the agent to retain and process past observations, mitigating the impact of noise and variations in geomagnetic field patterns and ensuring coherent navigation decisions over time. This finding underscores a broader principle: memory mechanisms are essential for robust navigation across diverse modalities and environments. We further validated PPO-LSTM’s deployment feasibility on our edge device, demonstrating its practical applicability for resource-constrained platforms.

Our study opens several avenues for future research. Exploring advanced memory architectures, such as attention mechanisms or transformers, could further enhance the agent’s ability to manage long-horizon trajectories and complex environments. Integrating complementary sensor modalities—such as inertial ones—might improve adaptability. Moreover, the principles of bio-inspired geomagnetic navigation, which leverage the Earth’s magnetic field for orientation, hold considerable potential for adaptation to localized scenarios, like indoor navigation. In GPS-denied settings, such as underwater environments, or aerial navigation for drones in regions with minimal geomagnetic interference, localized magnetic landmarks could be introduced to replicate natural navigation cues, thereby providing a robust alternative for precise localization and pathfinding. By pursuing these avenues, future work can build upon our findings to enhance the reliability and efficiency of autonomous navigation under uncertainty.

## Figures and Tables

**Table 1:** Hyperparameters for PPO-LSTM algorithm in simulation.

Hyperparameter	Value
Normalize observations	True
Number of parallel environments	1
Total training time steps	1,000,000
Batch size for updates	512
Steps per environment per update	2048
Discount factor (γ)	0.995
Learning rate	0.00010881173007479382
Entropy coefficient	3.651742693016306 × 10^−6^
Clip range	0.2
Number of epochs per update	20
GAE lambda (λ)	0.95
Maximum gradient norm	0.8
Value function coefficient	0.46198070763807675
Initial log standard deviation	−2
Orthogonal initialization	False
Activation function	ReLU
**Network Architecture**	
Feature extractor type	LSTM
Feature extractor hidden layers	1
Feature extractor hidden layer size	64
Policy network type	MLP
Policy network hidden layers	2
Policy network hidden layer sizes	[64, 64]
Critic network type	MLP
Critic network hidden layers	2
Critic network hidden layer sizes	[64, 64]

**Table 2:** SR and SPL of PPO and PPO-LSTM before and after adding noise.

Algorithm	Without Noise		With Noise	
	SR (%)	SPL (%)	SR (%)	SPL (%)
PPO	94.7	74.41856	76.3	57.92835
PPO-LSTM	**95.7**	**84.89380**	**79.3**	**65.88631**

**Table 3:** Trajectory smoothness and heading deviation metrics (MSE and MAE) for PPO-LSTM, PPO, MPSC, and DE across six tasks.

Task	Algorithm	Smoothness		Heading Deviation	
		MSE	MAE	MSE	MAE
Task 1	PPO-LSTM	0.2408	0.3558	0.1049	0.2562
	PPO	0.0481	0.1247	0.1927	0.3769
	MPSC	5.3887	1.0685	0.1624	0.2558
	DE	0.8259	0.4696	0.7740	0.6569
Task 2	PPO-LSTM	0.0042	0.0298	0.0360	0.1737
	PPO	0.0250	0.0932	0.3091	0.3651
	MPSC	0.8102	0.5781	0.0560	0.1599
	DE	0.4436	0.4626	0.1554	0.2413
Task 3	PPO-LSTM	0.0788	0.1952	0.0676	0.2119
	PPO	0.0733	0.1405	0.2668	0.4380
	MPSC	9.4671	1.7805	0.5978	0.4016
	DE	0.1252	0.2812	0.3787	0.4660
Task 4	PPO-LSTM	0.0080	0.0465	0.0436	0.1509
	PPO	0.0134	0.0957	0.2462	0.4671
	MPSC	1.7252	0.7933	0.1417	0.2821
	DE	0.1994	0.3035	0.5704	0.5906
Task 5	PPO-LSTM	0.0366	0.0768	0.0431	0.1768
	PPO	0.2327	0.3202	0.4567	0.6101
	MPSC	3.1330	1.1738	0.1903	0.3451
	DE	4.5133	1.0672	1.0579	0.7055
Task 6	PPO-LSTM	0.0063	0.0418	0.0987	0.2699
	PPO	0.0634	0.1254	0.1131	0.3209
	MPSC	3.4181	1.1068	0.5582	0.5171
	DE	0.7108	0.3656	0.7149	0.6387
**Ave.**	PPO-LSTM	**0.0625**	**0.1243**	**0.0657**	**0.2066**
	PPO	0.0760	0.1499	0.2641	0.4297
	MPSC	3.9904	1.0835	0.2844	0.3269
	DE	1.1364	0.4916	0.6086	0.5498

## Data Availability

Datasets available on request from the authors.
